# *Kcna3* Deficiency Promotes Renin-Associated Hypertension Through Ca^2+^-Dependent AKT–PKA–CREB Signaling

**DOI:** 10.3390/biom16091262

**Published:** 2026-08-31

**Authors:** Ye Wang, Shiyun Sun, Yuhan Zhang, Guoqing Li, Yunlong Xu, Yingxue Shi, Pedro A. Jose, Zhiwei Yang, Xing Liu, Xiaoliang Jiang

**Affiliations:** 1 Institute of Laboratory Animal Science, Chinese Academy of Medical Sciences (CAMS) & Comparative Medicine Center, Peking Union Medical College (PUMC), National Human Diseases Animal Model Resource Center, NHC Key Laboratory of Comparative Medicine, National Center of Technology Innovation for Animal Model; State Key Laboratory of Respiratory Health and Multimorbidity, Panjiayuan Nanli, Chaoyang District, Beijing 100021, China; 2 Institute of Clinical Medical Sciences, China-Japan Friendship Hospital, Chinese Academy of Medical Sciences & Peking Union Medical College, Beijing 100021, China; 3 Basic Medical College, Hebei North University, Zhangjiakou 075000, China; 4 Department of Pharmacology and Physiology, The George Washington University School of Medicine & Health Sciences, Washington, DC 20052, USA; 5 Division of Kidney Diseases & Hypertension, Department of Medicine, The George Washington University School of Medicine & Health Sciences, Washington, DC 20052, USA

**Keywords:** *Kcna3*, hypertension, renin, calcium signaling, CREB, PI3K–AKT pathway, RAAS

## Abstract

Hypertension is an important risk factor for cardiovascular and renal diseases, yet the mechanisms linking ion channel dysfunction to hypertension remain poorly understood. The voltage-gated potassium channel Kv1.3 (encoded by *Kcna3*) regulates membrane potential, but its role in the pathogenesis of hypertension remains unclear. In this study, we employed *Kcna3* knockout (KO) mice, transcriptomic profiling, and pharmacological inhibition to investigate the role of Kv1.3. *Kcna3*-deficient mice showed increased blood pressure and renal fibrosis. Transcriptomic profiling showed activation of the renin–angiotensin–aldosterone system (RAAS), with increased renin expression in both *Kcna3*-deficient mice and Kv1.3 inhibitor-treated cells. Mechanistically, loss of Kv1.3 increased intracellular Ca^2+^ accumulation, activating the phosphoinositide 3-kinase (PI3K)-AKT and protein kinase A (PKA) pathways, leading to cAMP response element binding protein (CREB) phosphorylation and renin upregulation. Pharmacological inhibition of Ca^2+^ signaling or PKA reduced CREB phosphorylation and renin expression, confirming a causal signaling cascade. Thus, Kv1.3 links membrane excitability to RAAS activation via a Ca^2+^-dependent AKT–PKA–CREB signaling axis and represents a potential therapeutic target for hypertension and associated renal injury.

## 1. Introduction

Hypertension, a global health burden, is an important risk factor for cardiovascular and chronic kidney diseases [1]. Hypertension is characterized by dysregulation of the renin–angiotensin–aldosterone system (RAAS), which contributes to the regulation of blood pressure and fluid homeostasis [2,3]. In particular, renin, a rate-limiting enzyme of the RAAS, is regulated at the transcriptional and post-transcriptional levels [2,4]. Previous studies have shown that, in addition to classical hormonal regulation, intracellular signaling pathways contribute to the modulation of renin expression. However, the upstream factors linking cellular electrophysiological states to RAAS activation are unclear [5,6].

Ion channels regulate membrane excitability and cellular signaling. For instance, voltage-gated potassium channels regulate membrane potential and ion homeostasis [7,8]. Kv1.3, encoded by the *Kcna3* gene, is a delayed rectifier potassium channel widely expressed in immune and non-immune tissues. Kv1.3 functions to regulate K^+^ efflux and promote membrane repolarization [9,10]. Kv1.3 dysregulation contributes to the pathogenesis of metabolic disorders, inflammation, and autoimmune diseases [11,12]. However, the role of Kv1.3 in cardiovascular and renal physiology, especially in regulating blood pressure remains unclear [13,14]. Ion channel activity can modulate intracellular Ca^2+^ dynamics by modulating membrane potential. This suggests that the function of Kv1.3 is correlated with Ca^2+^-dependent signaling pathways [8,15].

Intracellular Ca^2+^ acts as a critical second messenger regulating multiple signaling cascades, including the PI3K-AKT and cAMP-PKA pathways [16,17]. These pathways converge on the transcription factor cAMP response element-binding protein (CREB), which has been reported to regulate *REN* transcription. The phosphorylation of CREB enhances its transcriptional activity and promotes renin expression, contributing to RAAS activation [18,19]. The downstream regulation of renin by Creb1 has been extensively studied. However, the upstream mechanisms that trigger Creb1 activation in renal cells, especially those linked to ion channel function, remain undefined [20,21].

This study hypothesized that Kv1.3 regulates blood pressure by modulating intracellular Ca^2+^ signaling and downstream transcriptional pathways controlling renin expression. To test this hypothesis, bioinformatics analysis, genetic manipulation, and pharmacological approaches were used to examine the role of Kv1.3 in hypertension. *Kcna3* deficiency did not alter its transcript levels under hypertensive conditions but promoted intracellular Ca^2+^ accumulation. This led to the activation of the PI3K-AKT and PKA pathways and the upregulation of p-Creb1 and renin. These findings indicate that Kv1.3 is a critical link between membrane excitability and endocrine regulation of blood pressure, providing new insights into the pathogenesis of hypertension and potential therapeutic targets.

## 2. Materials and Methods

### 2.1. Animal Studies and Ethics Statement

All animal experiments were conducted in compliance with institutional guidelines and in accordance with international standards for laboratory animal care. The study protocol was reviewed and approved by the Animal Care and Use Committee of the Institute of Laboratory Animal Science, Chinese Academy of Medical Sciences and Peking Union Medical College (Approval No. YZW23003).

*Kcna3* global knockout (KO) mice (hereafter referred to as *Kcna3*-deficient mice) on a *C57BL/6J* background were generated through CRISPR/Cas9-mediated deletion in the coding region of the *Kcna3* gene and were purchased from Beijing V-Solid Biotech Co., Ltd (Beijing, China). Age- and sex-matched wild-type (WT) littermates served as controls. Male and female mice aged 10–14 weeks were included in all experiments unless stated otherwise. For assessment of hypertension and renal injury, mice were allocated to two groups: WT (n = 8–10) and *Kcna3* KO (n = 8–10). All animals were housed under specific pathogen–free conditions at a controlled temperature (22–24 °C) and a 12-h light/dark cycle, and had unrestricted access to standard chow and water.

Blood pressure recording, tissue collection, and subsequent histological and molecular analyses were conducted by investigators blinded to genotype. At the end of the experiment, mice were deeply anesthetized. Blood samples were obtained from the inferior vena cava with a sterile syringe and needle, and immediately transferred into tubes containing dipotassium ethylenediaminetetraacetate (K_2_-EDTA) as an anticoagulant. Plasma was isolated by centrifugation at 3000× *g* for 15 min at 4 °C. Plasma samples were sent to a certified third-party provider (Shanghai Xinfan Biotechnology Co., Ltd., Shanghai, China) for quantification of renin, angiotensin II, and aldosterone using commercial ELISA kits (renin: Mouse renin ELISA Kit, Cat. No. XFM21087; angiotensin II: Mouse Angiotensin II ELISA Kit, Cat. No. XFM21329; aldosterone: Mouse Aldosterone ELISA Kit, Cat. No. XFM21032; all from GEMIG, Nanjing, China). According to the manufacturer’s datasheet, the renin ELISA kit detects active renin with no cross-reactivity with prorenin. Plasma levels of aspartate aminotransferase (AST), creatine kinase-MB (CK-MB), creatine kinase (CK), and lactate dehydrogenase (LDH) were also quantified by the same provider using commercial assay kits (AST: Cat. No. B2009; CK-MB: Cat. No. B2030; CK: Cat. No. B2029; LDH: Cat. No. B2028; all from Bioteke, Beijing, China) on an automated biochemical analyzer. After blood collection, mice were transcardially perfused with ice-cold phosphate-buffered saline (PBS, pH 7.4). Kidneys, adrenal glands, heart, and liver were promptly harvested; portions of each tissue were snap-frozen in liquid nitrogen for molecular biology analyses, and the remaining tissues were fixed in 4% paraformaldehyde for histological and immunofluorescence analyses.

### 2.2. Public Dataset Analysis

The microarray gene expression dataset GSE41453 was retrieved from the Gene Expression Omnibus (GEO) database. This dataset contains renal tissue samples from normotensive Wistar–Kyoto (WKY) rats and spontaneously hypertensive rats (SHRs), profiled on the Affymetrix Rat Genome 230 2.0 Array platform. Raw expression data (CEL files) were processed with the robust multi-array average (RMA) algorithm for background correction, quantile normalization, and log_2_ transformation. Probe-level intensities were summarized to gene-level expression values using the corresponding platform annotation files. Genes with low expression were excluded by retaining only those with an expression level above the 20th percentile in at least 50% of the samples.

Differential expression analysis between the SHRs and WKY groups was conducted using an empirical Bayes moderated *t*-test. The resulting *p*-values were corrected for multiple testing using the Benjamini–Hochberg false discovery rate (FDR) method. Genes with |log_2_ fold change| > 1 and adjusted *p*-value < 0.05 were considered significantly differentially expressed. Data visualization was performed in R version 4.5.0 (R Core Team, Vienna, Austria). Volcano plots were generated to show the overall distribution of differentially expressed genes, with significantly upregulated and downregulated genes indicated in distinct colors. Boxplots of Kcna3 expression were generated to compare normalized expression levels between the WKY and SHRs groups.

### 2.3. Blood Pressure Measurement

Continuous ambulatory blood pressure was monitored in conscious, freely moving mice using an implantable radiotelemetry system (Stellar_PTA-XS, TSE Systems GmbH, Berlin, Germany). Before surgery, each transmitter was calibrated following the manufacturer’s instructions. Mice were anesthetized with isoflurane (2–3% in oxygen), and a midline cervical incision was made to expose the left common carotid artery. The catheter tip of the telemetry probe was inserted into the carotid artery and advanced toward the thoracic aorta, secured by sutures, and the transmitter body was positioned subcutaneously along the right flank. Carprofen (5 mg/kg, s.c.) was administered as perioperative analgesia. Mice were allowed a recovery period of at least 7 days post-surgery before data acquisition. After recovery, SBP and DBP were recorded continuously for 72 h per weekly monitoring session over a total experimental period of 3 weeks, with freely moving mice under standard housing conditions. Recorded parameters were averaged over the entire recording period for each animal. Telemetry blood pressure data were analyzed by two-way ANOVA (time × genotype) to assess genotype effects and time-dependent trends. A significant genotype effect was confirmed for all parameters, with no significant time × genotype interaction, indicating a stable hypertensive phenotype.

To independently confirm the telemetry measurements, direct arterial blood pressure was measured by carotid artery cannulation in anesthetized mice. Mice were anesthetized with isoflurane (2% in oxygen) and placed on a heated pad to maintain body temperature at 37 °C. The right common carotid artery was exposed and then cannulated with a PE-10 catheter filled with heparinized saline (100 U/mL), connected to a calibrated pressure transducer. After a 15 min stabilization period, arterial pressure waveforms were recorded continuously for 20 min using a PowerLab data acquisition system (AD Instruments, Sydney, Australia) and LabChart 8 software. SBP and DBP were derived from the recorded waveforms and averaged over the stable recording period. At the end of the measurement, animals were euthanized while still under deep anesthesia.

### 2.4. Transthoracic Echocardiography

Transthoracic echocardiography was performed on WT and *Kcna3* KO mice under light isoflurane anesthesia. M-mode images were obtained from the parasternal short-axis view at the level of the papillary muscles. Left ventricular ejection fraction (EF), fractional shortening (FS), and left ventricular posterior wall thickness (LVPW) were quantified from the M-mode tracings and averaged over three consecutive cardiac cycles.

### 2.5. Transcriptomic and Statistical Analysis

Kidney and adrenal gland samples were subjected to bulk RNA sequencing (RNA-seq) at OE Biotech (Shanghai, China). Total RNA was extracted, and mRNA libraries were constructed. Paired-end sequencing was performed on an Illumina platform. For the kidney transcriptome, clean reads were mapped to the reference genome using HISAT2, and transcript abundance (as transcripts per million, TPM) was estimated and normalized using feature counts. Sample clustering was assessed by principal component analysis (PCA). DESeq2 was used to identify differentially expressed genes between WT and KO groups, using cutoffs of |log_2_ fold change| > 1 and adjusted *p* < 0.05. Expression patterns of the differentially expressed genes were visualized in heatmaps using Z-score-transformed values. Functional annotation was conducted using Gene Ontology (GO) and KEGG pathway enrichment analyses with clusterProfiler, while Gene Set Enrichment Analysis (GSEA) was used to detect pathway-level shifts using MSigDB gene sets (FDR < 0.25). Group comparisons were conducted using unpaired two-tailed Student’s *t*-test, and a *p* value below 0.05 was considered statistically significant. All results are presented as mean ± SEM.

### 2.6. Measurement of Tissue Total Calcium Content

Kidney tissues were homogenized in ice-cold lysis buffer and centrifuged to remove cellular debris. Total calcium concentration in the supernatant was quantified using a commercial calcium assay kit (S1063S, Beyotime, Shanghai, China) following the manufacturer’s instructions. Calcium levels were normalized to total protein concentration determined by a BCA assay. Results are reported as μg calcium per mg protein.

### 2.7. Histological Analysis

Kidney tissues were fixed in 4% paraformaldehyde at 4 °C for 24 h, dehydrated through a graded ethanol series, cleared in xylene, and embedded in paraffin. For general morphological assessment, sections were deparaffinized, rehydrated, and stained with hematoxylin and eosin (H&E). Collagen deposition and fibrosis were assessed by Masson’s trichrome staining. Stained sections were examined by light microscopy, and representative images were acquired at 40× magnification. The area of collagen deposition (blue staining) was quantified with ImageJ software (Fiji, ImageJ 2.16.0/1.54p; National Institutes of Health, Bethesda, MD, USA) and reported as fibrotic area per unit tissue area.

### 2.8. RNA Extraction and Quantitative Real-Time PCR (qPCR)

Total RNA was isolated from tissues with TRIzol reagent (Invitrogen, CA, USA), and RNA concentration and purity were assessed on a NanoDrop spectrophotometer (Thermo Fisher Scientific, Waltham, MA, USA). First-strand cDNA was synthesized from 1 μg of total RNA using the High-Capacity cDNA Reverse Transcription Kit (Applied Biosystems, Foster City, CA, USA). Quantitative real-time PCR (qRT-PCR) amplification was performed using SYBR Green PCR Master Mix (RR820A, Takara, Kusatsu, Shiga, Japan) on a StepOnePlus Real-Time PCR System (Applied Biosystems, Waltham, MA, USA). Target gene expression was determined by the 2^^−ΔΔCt^ method, with GAPDH serving as the endogenous control. The primer sequences for Kcna3 were as follows:

Forward, 5′-ATCCTCTACTACTACCAGTCCG-3′;

Reverse, 5′-CCTCATCCTCACGGAACTTTTC-3′.

### 2.9. Western Blot Analysis

Whole-cell protein lysates were obtained from cells using RIPA buffer supplemented with 100× protease inhibitor cocktail (Sigma-Aldrich, St. Louis, MO, USA) and 10× PhosSTOP phosphatase inhibitor (Roche, Basel, Switzerland). Protein concentration was quantified using the enhanced BCA Protein Assay Kit (Beyotime). Equal amounts of protein were resolved by SDS-PAGE and electron transferred onto nitrocellulose membranes. Nonspecific binding was blocked by incubating membranes with 5% (*w*/*v*) non-fat dry milk or BSA in TBST for 1 h at room temperature. Membranes were subsequently probed overnight at 4 °C with the following primary antibodies:

Kcna3 (14079-1-AP, Proteintech, Rosemont, IL, USA, Rabbit, 1:1000 dilution)

Renin (14291-1-AP, Proteintech, Rabbit, 1:1000 dilution; note: the antibody detected a single 45 kDa band, interpreted as total renin-related protein)

Mmp2 (10373-2-AP, Proteintech, Rabbit, 1:1000 dilution)

Mmp9 (10375-2-AP, Proteintech, Rabbit, 1:1000 dilution)

Pik3ca (bs-2067R, Bioss, Rabbit, 1:1000 dilution)

phospho-Pik3ca (Tyr317) (bs-5570R, Bioss, Rabbit, 1:1000 dilution)

Akt (10176-2-AP, Proteintech, Rabbit, 1:1000 dilution)

phospho-Akt (Ser473) (66444-1-Ig, Proteintech, Rabbit, 1:1000 dilution)

Creb1 (12208-1-AP, Proteintech, Rabbit, 1:2000 dilution)

Phospho-Creb1 (Ser133) (28792-1-AP, Proteintech, Rabbit, 1:1000 dilution)

GAPDH (HRP-60004, Proteintech, 1:10,000 dilution)

After thorough washing, the membranes were incubated with horseradish peroxidase (HRP)-conjugated secondary antibodies for 1 h at room temperature. Immunoreactive bands were detected using enhanced chemiluminescence (ECL) reagent and captured on a Tanon 5500 Chemiluminescent Imaging System (Tanon, Shanghai, China). Densitometric analysis was performed using ImageJ software, and band intensities were normalized to the GAPDH loading control.

### 2.10. Immunofluorescence Staining

Immunofluorescence staining was conducted on paraffin-embedded kidney sections (4 μm thick). After dewaxing in xylene and rehydration through a graded ethanol series (100%, 95%, 80%, and 70%), antigen retrieval was conducted by microwaving sections in 10 mM citrate buffer (pH 6.0) at 95–100 °C for 15 min, and then allowed to cool to room temperature. The sections were subsequently permeabilized with 0.1% Triton X-100 (Sigma) in PBS for 10 min and blocked with 2% goat serum (Sigma) for 1 h at room temperature. Primary antibody incubation was performed overnight at 4 °C using the following antibodies diluted in blocking solution: anti-Kcna3 (14079-1-AP, Proteintech, rabbit, 1:200), anti-renin (14291-1-AP, Proteintech, rabbit, 1:500), anti-phospho-Creb1 (Ser133) (28792-1-AP, Proteintech, rabbit, 1:800), and anti-Aqp2 (sc-515770, Santa Cruz Biotechnology, Dallas, TX, USA mouse, 1:200). After three 10 min washes with 0.1% PBST (PBS containing 0.1% Tween 20), sections were incubated with fluorophore-conjugated secondary anti-bodies (Alexa Fluor 488 or Alexa Fluor 594) for 1 h at room temperature in the dark. Sections were then mounted with DAPI-containing mounting medium (ZLI-9557, ZSGB-BIO, Beijing, China). Images were acquired using a Fluoview FV 100 confocal microscope (Olympus, Tokyo, Japan) and processed with ImageJ software.

### 2.11. Cell Culture and Pharmacological Treatments

The mouse renal collecting duct M-1 cell line (SV40-transformed) was purchased from Shangcheng Beinachuanglian Biotechnology Co., Ltd.(Shangcheng, Henan, China) and was maintained in DMEM/F-12 medium supplemented with 0.005 mM dexamethasone, 5% fetal bovine serum (FBS), and 1% penicillin-streptomycin at 37 °C in a humidified atmosphere with 5% CO_2_. Cells were seeded in 6-well plates or 24-well plates containing coverslips and grown to 70–80% confluence before pharmacological treatment.

The following pharmacological inhibitors were employed: PAP-1 Cat# TQ0179, TargetMol, Boston, MA, USA (10 nM), a selective Kv1.3 channel inhibitor; BAPTA-AM (Cat# HY-100545, MedChemExpress, Monmouth Junction, NJ, USA, 10 μM), an intracellular Ca^2+^ chelator; and H89 (Cat# HY-15979, MedChemExpress, 10 μM), a PKA inhibitor. Each inhibitor was dissolved in DMSO, and control cells received an equal volume of DMSO (final concentration < 0.1%).

Cells were assigned to the following treatment groups: (1) vehicle control (DMSO); (2) PAP-1 alone; (3) PAP-1 + BAPTA-AM (pretreated with BAPTA-AM for 40 min before PAP-1 stimulation); and (4) PAP-1 + H89 (pretreated with H89 for 1 h before PAP-1 stimulation). Following 24 h of treatment, cells were collected for protein extraction and western blot analysis, or fixed for subsequent immunofluorescence staining.

### 2.12. Statistical Analysis

Data are presented as mean ± SEM. Normality of data distribution was evaluated using the Shapiro–Wilk test prior to parametric analyses. All statistical tests were performed using GraphPad Prism version 10.1.2 (GraphPad Software, Boston, MA, USA). Two-group differences were evaluated by unpaired two-tailed Student’s *t*-test, whereas multi-group comparisons were performed using one-way or two-way analysis. Statistical significance was set at *p* < 0.05.

## 3. Results

### 3.1. Kcna3 Deficiency Alters the Renal Transcriptional Profiles

**Figure 1:**
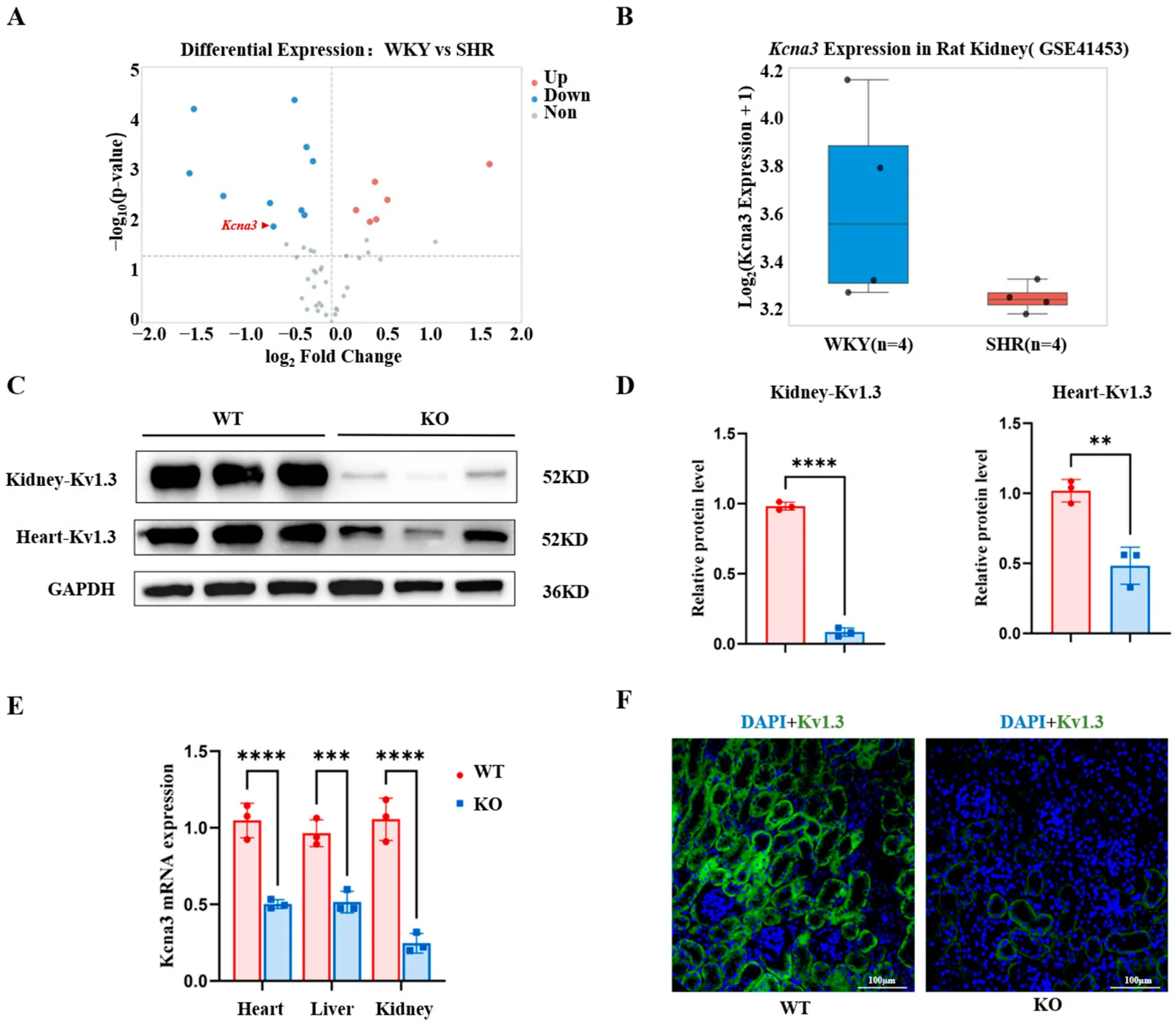
*Kcna3* expression is not altered in hypertension. (**A**) Volcano plot revealed that the renal levels of *Kcna3* were not significantly different between Wistar Kyoto (WKY) rats and spontaneously hypertensive rats (SHRs) (GSE41453). Dashed lines indicate the thresholds for statistical significance (*p* < 0.05, horizontal) and fold change (|log_2_ fold change| > 1, vertical). (**B**) Boxplot of *Kcna3* expression levels in WKY rats and SHRs. (**C**) Western blotting analysis of Kv1.3 expression in the renal and cardiac tissues of wild-type (WT) and *Kcna3* knockout (KO) mice. (**D**) Quantification of Kv1.3 protein levels in renal and cardiac tissues of WT and *Kcna3* KO mice. (**E**) Quantitative real-time polymerase chain reaction analysis of *Kcna3* mRNA levels in different tissues of WT and *Kcna3* KO mice. (**F**) Representative immunofluorescence staining images of Kv1.3 in the kidneys of WT and *Kcna3* KO mice. Scale bar = 100 μm. In all bar graphs, blue bars denote KO and red bars denote WT. Data are presented as mean ± standard error of the mean. ** *p* < 0.01, *** *p* < 0.001, **** *p* < 0.0001 (unpaired Student’s *t*-test). Original Western blot images are provided in the Supplementary Materials.

The role of *Kcna3* in hypertension was examined by analyzing the public dataset GSE41453. The GSE41453 dataset contains transcriptomic data from renal tissues of normotensive Wistar Kyoto (WKY) rats and spontaneously hypertensive rats (SHRs). Differential expression analysis revealed that *Kcna3* expression was not significantly different between WKY rats and SHRs (Figure 1A). Consistently, quantitative analysis confirmed similar *Kcna3* expression levels between WKY rats and SHRs (Figure 1B). This indicates that hypertension in *Kcna3*-deficient mice is unlikely to result from transcriptional upregulation of *Kcna3* itself, supporting further investigation of downstream signaling pathways.

Next, systemic *Kcna3* knockout (KO) mice were generated to evaluate the functional role of *Kcna3*. Western blotting analysis showed that the renal and cardiac levels of Kv1.3 were significantly downregulated in *Kcna3* KO mice (Figure 1C,D). Quantitative real-time polymerase chain reaction (qRT-PCR) analysis demonstrated that the *Kcna3* mRNA levels are downregulated in multiple tissues, especially in the kidney tissue (Figure 1E). Immunofluorescence staining revealed that the renal expression of Kv1.3 was abundant in wild-type (WT) mice but nearly absent in *Kcna3* KO mice (Figure 1F). Renal Kv1.3 protein was almost undetectable in *Kcna3* KO mice (Figure 1C,D), with densitometric analysis indicating a reduction of over 90% compared to WT controls, confirming effective gene disruption in the kidney—the primary organ of interest for this study. Residual expression was observed in cardiac tissues, possibly reflecting tissue-specific variability in CRISPR/Cas9 recombination efficiency. These data support successful gene modification at both transcriptional and protein levels (Figure 1C–F), with the greatest reduction observed in the kidney. The successful establishment of *Kcna3* KO mice provides a suitable model for subsequent functional studies.

### 3.2. Kcna3 Deficiency Promotes RAAS Activation, Hypertension, and Renal Injury

**Figure 2:**
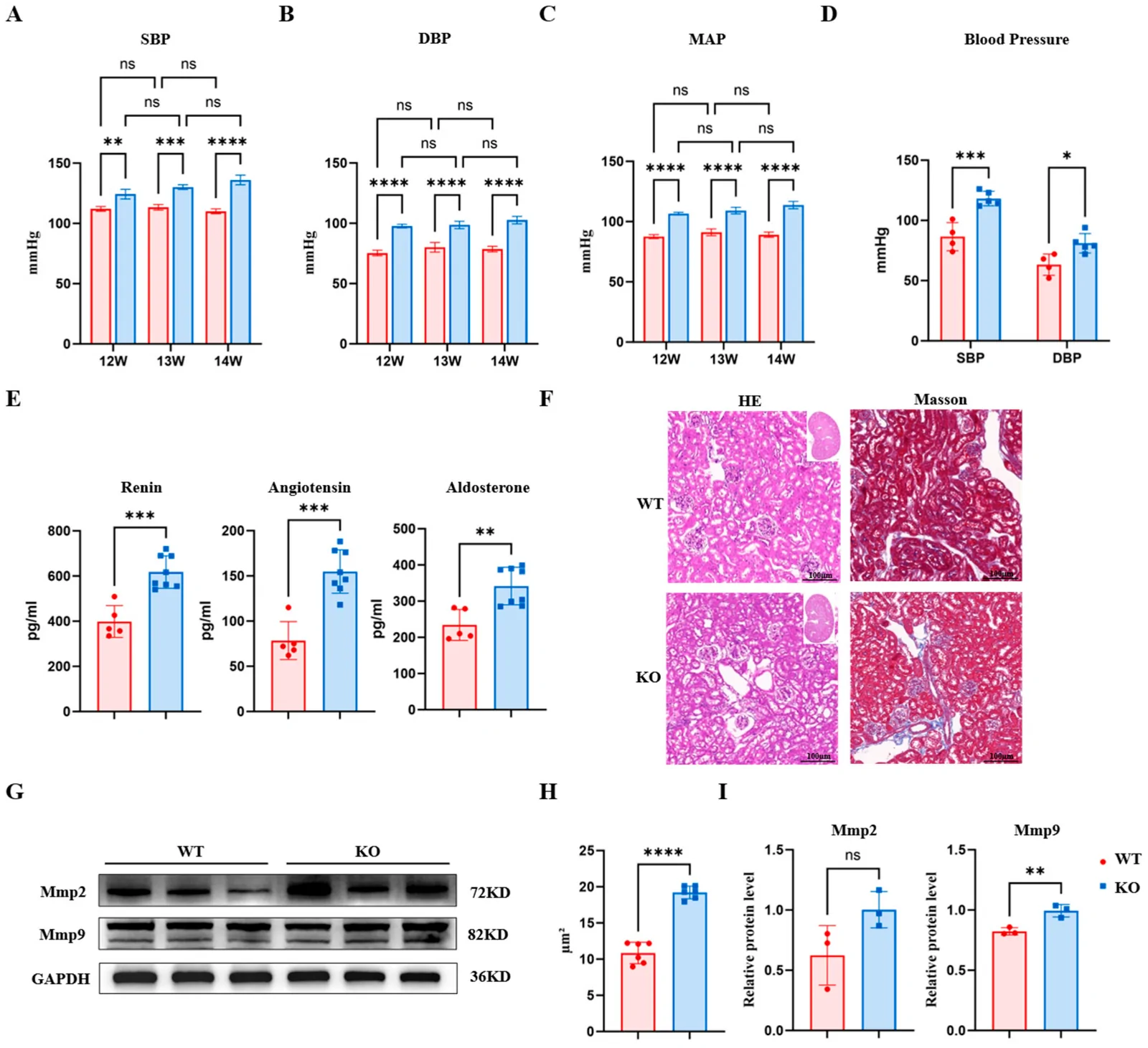
*Kcna3* deficiency promotes hypertension and renal injury. (**A**–**C**) Telemetric measurements of systolic blood pressure (SBP), diastolic blood pressure (DBP), and mean arterial pressure (MAP) in wild-type (WT) and *Kcna3* knockout (KO) mice across a 3-week observation period. Data were assessed by two-way ANOVA (time × genotype), revealing a significant genotype effect for SBP, DBP, and MAP (*p* < 0.001 for all) without a significant time × genotype interaction, supporting a stable hypertensive phenotype across the observation period. (**D**) Carotid artery-based measurement of arterial blood pressure confirmed elevated blood pressure in *Kcna3* KO mice. (**E**) The plasma levels of renin, angiotensin II, and aldosterone. (**F**) Representative images of renal sections subjected to hematoxylin and eosin (H&E) and Masson’s trichrome staining showing structural damage and increased collagen deposition. Scale bar = 100 μm. (**G**) Western blotting analysis of fibrosis-related proteins Mmp2 and Mmp9 in the kidney tissues. (**H**) Quantification of collagen-positive area. (**I**) Quantitative analysis of Mmp2 and Mmp9 levels. In all bar graphs, blue bars denote KO and red bars denote WT. Data are presented as mean ± SEM. Statistical significance was determined as described in the Methods section. * *p* < 0.05, ** *p* < 0.01, *** *p* < 0.001, **** *p* < 0.0001, ns, not significant. Original Western blot images are provided in the Supplementary Materials.

Telemetry monitoring showed that systolic blood pressure, diastolic blood pressure, and mean arterial pressure in *Kcna3* KO mice were significantly elevated compared with those in WT mice across the 3-week observation period, with no evident time-dependent trend within each genotype. Two-way ANOVA showed a significant genotype effect for SBP, DBP, and MAP (*p* < 0.001 for all), without a significant time × genotype interaction (Figure 2A–C). While the 5–8 mmHg SBP elevation was modest, it remained stable throughout the 3-week observation and was accompanied by consistent elevations in plasma renin, Ang II, and aldosterone. Analysis of circadian blood pressure patterns showed that the hypertensive phenotype in *Kcna3*-deficient mice was consistently present during both daytime (inactive phase) and nighttime (active phase) periods (Supplementary Figure S4). Consistently, carotid arterial pressure in *Kcna3*-deficient mice was higher than that in WT mice. These findings suggest that *Kcna3* deficiency induces a hypertensive phenotype (Figure 2D). Histological analysis indicated that *Kcna3* deficiency alters the renal structure. Hematoxylin and eosin (H&E) staining showed glomerular hypertrophy and tubular dilation, while Masson’s trichrome staining showed increased interstitial collagen deposition in *Kcna3*-deficient mice (Figure 2F), suggesting renal fibrosis. Consistently, western blotting analysis showed increased fibrosis-associated proteins, especially Mmp9, in the kidneys of *Kcna3* KO mice. The expression of Mmp2 was non-significantly upregulated in the kidney of *Kcna3*-deficient mice (Figure 2G,I). Thus, *Kcna3* deficiency increases blood pressure and promotes renal structural remodeling and fibrosis. Notably, echocardiographic analysis and circulating cardiac injury markers (CK, CK-MB, LDH, and AST) showed no significant differences between WT and Kcna3 KO mice (Figure S6), indicating that the hypertensive phenotype is unlikely to be secondary to primary cardiac dysfunction (Supplementary Figure S1).

### 3.3. Kcna3 Deficiency Activates the RAAS Pathway and Upregulates Renin Expression

**Figure 3:**
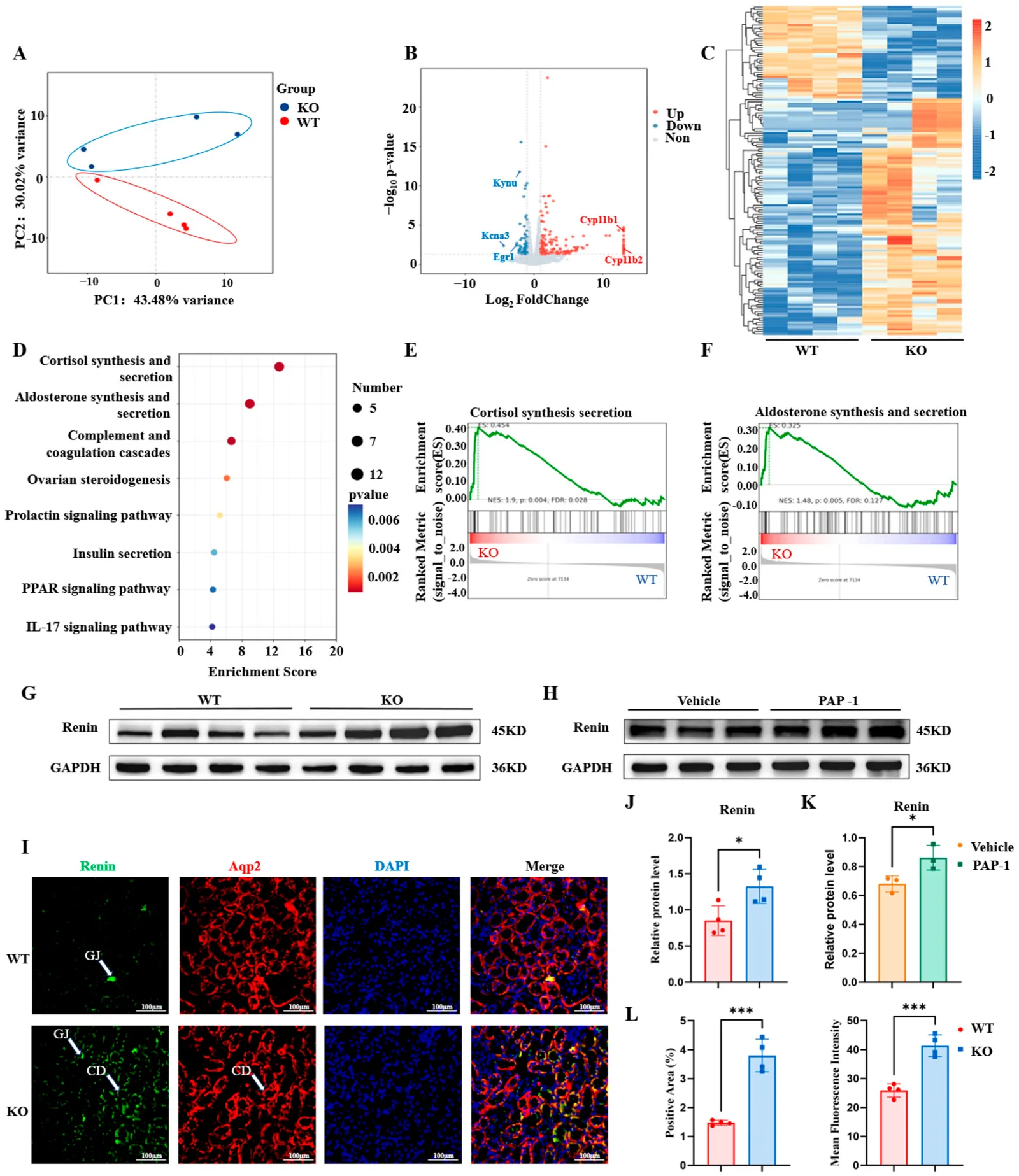
*Kcna3* deficiency activates renin–angiotensin–aldosterone system (RAAS)-related pathways and promotes renin expression. (**A**) Principal component analysis (PCA) of renal transcriptomic profiles of wild-type (WT) and *Kcna3* knockout (KO) mice. (**B**) Volcano plot showing differentially expressed genes between WT and *Kcna3* KO mice. (**C**) Heatmap of hierarchical clustering of differentially expressed genes. (**D**) Kyoto Encyclopedia of Genes and Genomes (KEGG) pathway enrichment analysis of differentially expressed genes. (**E**,**F**) Gene set enrichment analysis (GSEA) showing the activation of cortisol synthesis and secretion and aldosterone synthesis and secretion pathways in *Kcna3* KO mice. (**G**) Western blotting analysis of renin expression in the kidney tissues of WT and *Kcna3* KO mice. (**H**) Western blotting analysis of renin expression after treatment with the Kv1.3 inhibitor 5-(4-phenoxybutoxy) psoralen (PAP-1). (**I**) Representative immunofluorescence staining images of renin (green) and Aqp2 (red) in the renal sections. Nuclei are stained with 4′,6-diamidino-2-phenylindole (DAPI) (blue). Scale bar = 100 μm. (**J**,**K**) Quantitative analysis of renin levels. (**L**) Quantification of renin fluorescence intensity. In all bar graphs, blue bars denote KO, red bars denote WT, yellow denotes Vehicle, and green denotes PAP-1. Data are presented as mean ± standard error of the mean. * *p* < 0.05, *** *p* < 0.001 (unpaired Student’s *t*-test). Original Western blot images are provided in the Supplementary Materials.

The kidney tissues of WT and *Kcna3*-deficient mice were subjected to transcriptomic analysis. Principal component analysis (PCA) showed that the transcriptome of WT and *Kcna3* KO mice exhibited distinct clustering (Figure 3A). Among these differentially expressed genes, *Kcna3* was downregulated, whereas several steroid metabolism-related genes were markedly upregulated in *Kcna3* KO mice (Figure 3B). Hierarchical clustering further supported consistent gene expression patterns in the two groups (Figure 3C). Pathway enrichment analysis showed that the differentially expressed genes were enriched in hormone-related pathways, including cortisol synthesis and secretion and aldosterone synthesis and secretion (Figure 3D). Gene set enrichment analysis (GSEA) supported activation of these pathways in *Kcna3* KO mice (Figure 3E,F). Similar transcriptional alterations were observed in the kidney cortex and adrenal gland tissues of *Kcna3* KO mice (Supplementary Figures S2 and S3). These findings indicate the systemic involvement of RAAS-related pathways.

Given the role of the RAAS in blood pressure regulation, renin expression was evaluated. Western blotting analysis showed that the renal renin levels in *Kcna3*-deficient mice were significantly upregulated when compared with those in WT mice (Figure 3G,J). Additionally, the pharmacological inhibition of Kv1.3 using 5-(4-phenoxybutoxy)psoralen (PAP-1) upregulated renin expression (Figure 3H,K). This is consistent with a role of Kv1.3 in regulating renin expression. Immunofluorescence staining showed increased renin signal intensity in the renal tissues, predominantly distributed in tubular regions (CD-associated), with partial overlap with Aqp2-positive regions (Figure 3I,L). Hereafter, this pool is referred to as CD-associated renin to distinguish it from JG cell-derived circulating renin. These findings suggest that *Kcna3* deficiency activates RAAS-related pathways and promotes renin expression. Additionally, Kv1.3 may regulate renin expression through both transcriptional reprogramming and signaling pathway activation, contributing to the development of hypertension.

### 3.4. Kcna3 Deficiency Activates Ca^2+^-Dependent PI3K-AKT-CREB Signaling in the Kidney

**Figure 4:**
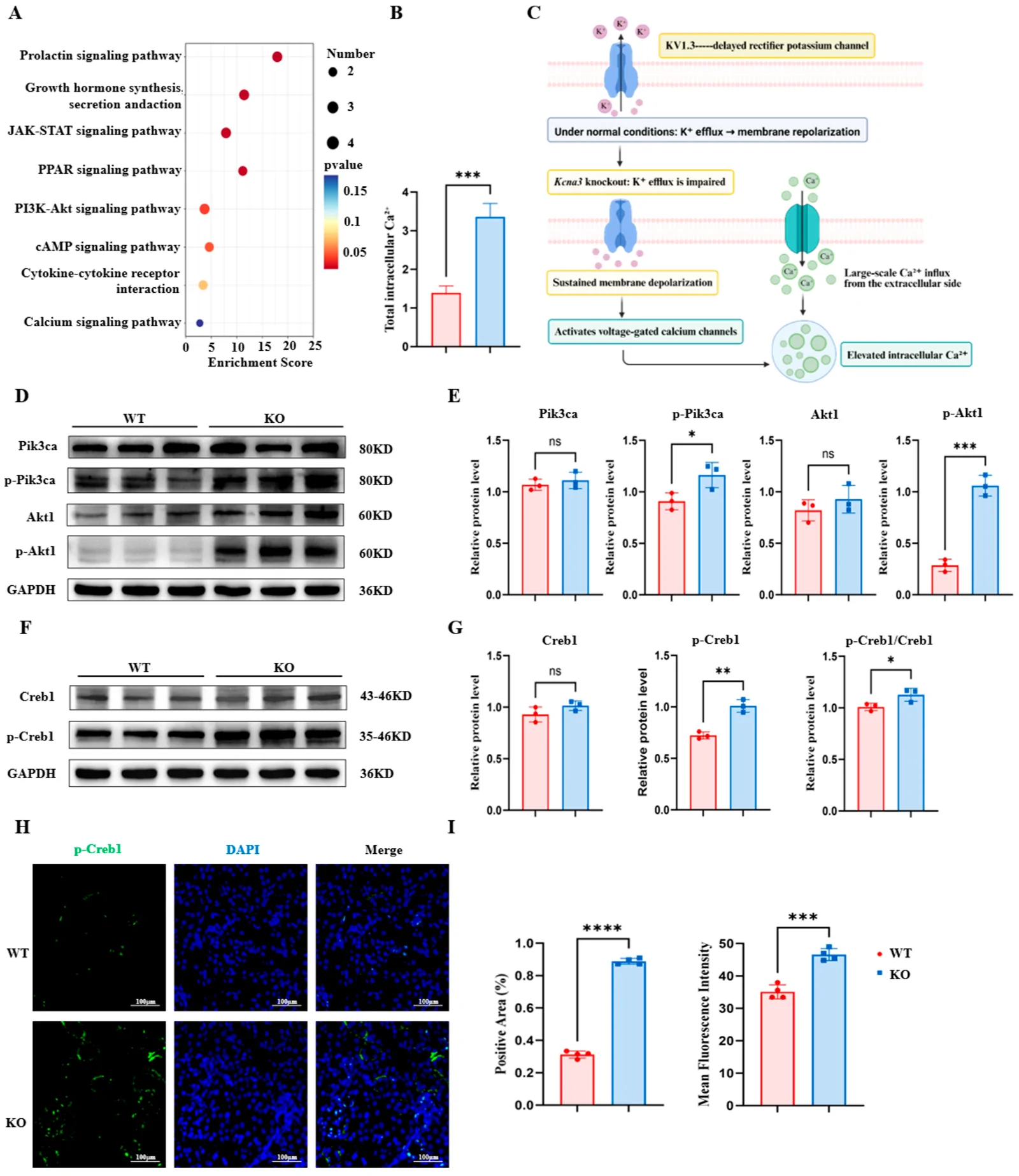
*Kcna3* deficiency activates Ca^2+^-dependent PI3K-AKT-CREB signaling in the kidney. (**A**) Kyoto Encyclopedia of Genes and Genomes (KEGG) pathway enrichment analysis of differentially expressed genes revealed the significant enrichment of PI3K-AKT, cAMP, and calcium signaling pathways. (**B**) Quantification of intracellular Ca^2+^ levels in wild-type (WT) and *Kcna3* knockout (KO) mice. (**C**) Schematic diagram of the proposed mechanism (Proposed mechanism): *Kcna3* deficiency impairs K^+^ efflux, leading to membrane depolarization and increased Ca^2+^ influx. (**D**) Western blotting analysis of Pik3ca, Akt1, p-Pik3ca, and p-Akt1 levels in the kidney tissues of WT and *Kcna3* KO mice. (**E**) Quantification of Pik3ca, Akt1, p-Pik3ca, and p-Akt1 levels. (**F**) Western blotting analysis of Creb1 and p-Creb1 in the kidney tissues of WT and *Kcna3* KO mice. (**G**) Quantification of Creb1 and p-Creb1 levels. (**H**) Representative immunofluorescence staining of p-Creb1 (green) in the kidney sections of WT and *Kcna3* KO mice. Nuclei were stained with 4′,6-diamidino-2-phenylindole (DAPI) (blue). Scale bar = 100 μm. (**I**) Quantification of p-Creb1-positive area and fluorescence intensity. In all bar graphs, blue bars denote KO and red bars denote WT. Data are presented as mean ± standard error of the mean. * *p* < 0.05, ** *p* < 0.01, *** *p* < 0.001, **** *p* < 0.000, ns, not significant. (unpaired Student’s *t*-test). Original Western blot images are provided in the Supplementary Materials.

The downstream signaling mechanisms through which *Kcna3* deficiency regulates renin expression were investigated. The transcriptomic data were subjected to pathway enrichment analysis. The pathways related to PI3K-AKT, cAMP, and calcium signaling were significantly enriched in *Kcna3*-deficient mice (Figure 4A), indicating the potential involvement of these pathways in *Kcna3*-mediated effects. As Kv1.3 has been reported to regulate membrane potential, this study examined the intracellular Ca^2+^ levels. The intracellular Ca^2+^ levels in *Kcna3* KO mice were significantly elevated compared with those in WT mice (Figure 4B). Previous studies have shown that Kv1.3 contributes to the maintenance of membrane potential by regulating K^+^ efflux. The loss or inhibition of Kv1.3 disrupts K^+^ conductance, resulting in membrane depolarization. This depolarized state facilitates the activation of voltage-gated Ca^2+^ channels, promoting Ca^2+^ influx.

The increased intracellular Ca^2+^ levels resulting from *Kcna3* deficiency can be attributed to enhanced Ca^2+^ entry after membrane depolarization (Figure 4C). Elevated intracellular Ca^2+^ can activate downstream pathways, including PI3K-AKT and CREB signaling (Figure 4C) [22,23].

The activation of the PI3K-AKT pathway. Western blotting analysis showed that the total Pik3ca and Akt1 levels were not significantly different between WT and *Kcna3* KO mice. In contrast, the renal p-Pik3ca and p-Akt1 levels were markedly upregulated in *Kcna3* KO mice (Figure 4D,E), consistent with activation of this pathway. Next, this study evaluated the signaling of CREB, a key transcription factor downstream of both PI3K-AKT and cAMP pathways. The total Creb1 levels were similar between WT and *Kcna3* KO mice. However, the p-Creb1 levels were significantly upregulated in *Kcna3* KO mice (Figure 4F,G), indicating enhanced transcriptional activity. Immunofluorescence staining confirmed increased renal p-Creb1 signal intensity in *Kcna3* KO mice (Figure 4H,I), indicating enhanced nuclear activation of Creb1 in situ. These findings are consistent with *Kcna3* deficiency-induced intracellular Ca^2+^ elevation, PI3K-AKT pathway activation, and p-Creb1 upregulation. These signaling events could contribute to the transcriptional upregulation of *REN*, linking *Kcna3* dysfunction to RAAS activation and hypertension.

### 3.5. Kv1.3 Regulates Renin Expression Through a Ca^2+^-Dependent Akt-Pka-Creb Signaling Axis

**Figure 5:**
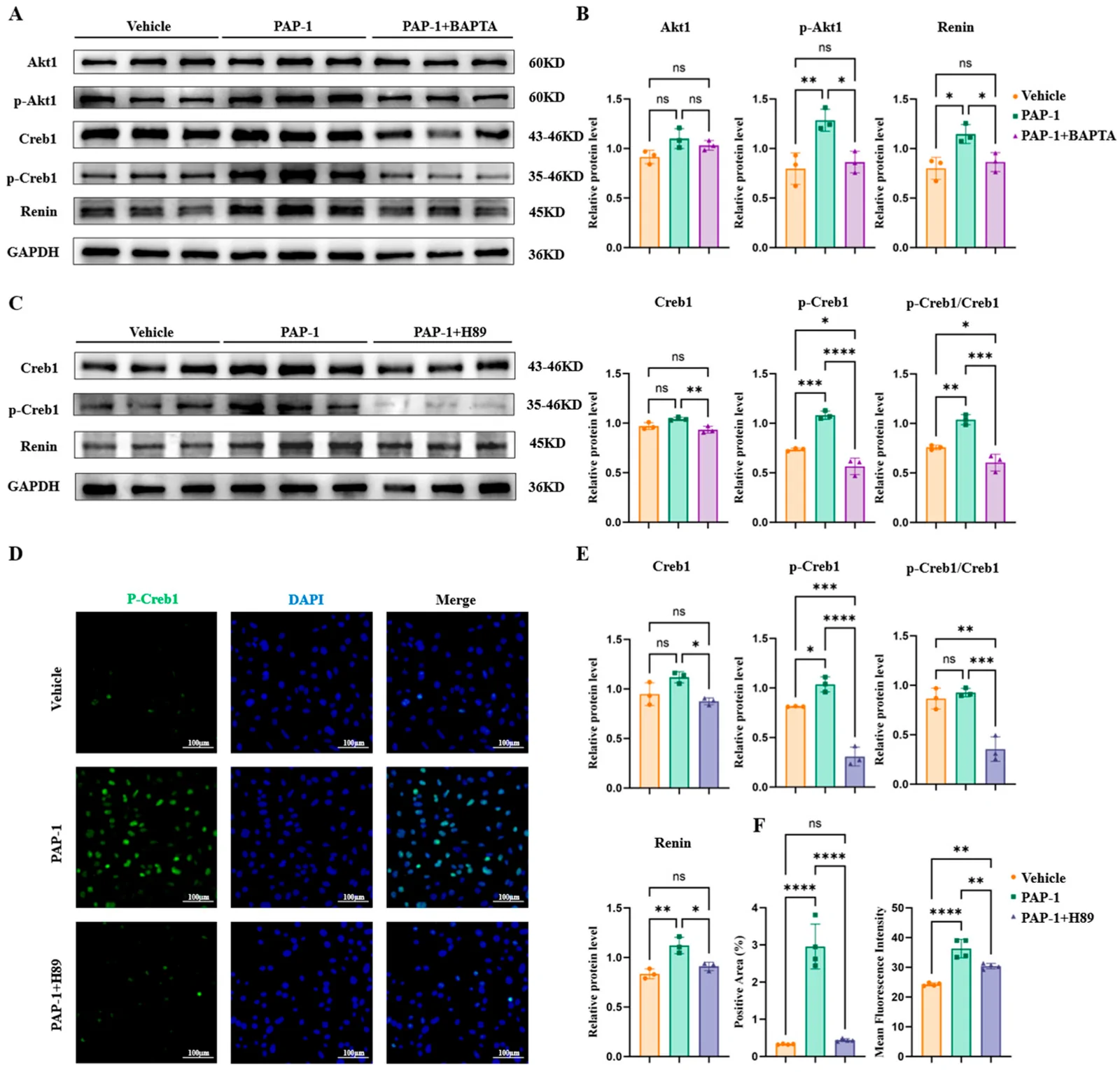
Pharmacological Kv1.3 inhibition promotes renin expression via Ca^2+^-dependent and PKA-dependent activation of AKT-CREB signaling. (**A**) Western blotting analysis of Akt1, p-Akt1, Creb1, p-Creb1, and renin levels in cells treated with vehicle, 5-(4-phenoxybutoxy) psoralen (PAP-1), or PAP-1 + the Ca^2+^ chelator 1,2-bis(o-aminophenoxy)ethane-N,N,N′,N′-tetraacetic acid (BAPTA). (**B**) Quantification of expression levels shown in (**A**). (**C**) Western blotting analysis of Creb1, p-Creb1, and renin in cells treated with vehicle, PAP-1, or PAP-1 + the protein kinase A (PKA) inhibitor H89. (**D**) Immunofluorescence staining of p-Creb1 (green) in different groups. Nuclei are stained with 4′,6-diamidino-2-phenylindole (DAPI) (blue). Scale bar = 100 μm. (**E**) Quantification of expression levels shown in (**C**). (**F**) Quantification of renin expression and p-Creb1 fluorescence intensities. In the bar graphs, yellow represents Vehicle, green represents PAP-1, light purple represents PAP-1+BAPTA, and dark purple represents PAP-1+H89. Data are presented as mean ± standard error of the mean. * *p* < 0.05, ** *p* < 0.01, *** *p* < 0.001, **** *p* < 0.0001, ns, not significant. (one-way analysis of variance, followed by a post hoc test). Original Western blot images are provided in the Supplementary Materials.

Next, the mouse cortical collecting duct cell line (M-1 cells) was subjected to pharmacological interventions. As shown in Figure 5A,B, treatment with PAP-1 significantly increased the p-Akt1, p-Creb1, and renin levels but did not affect the total Akt1 and Creb1 levels. Chelation of intracellular Ca^2+^ using 1,2-bis(o-aminophenoxy)ethane-N,N,N′,N′-tetraacetic acid (BAPTA) mitigated the PAP-1-induced upregulation of p-Akt1, p-Creb1, and renin levels, indicating that Ca^2+^ signaling is required for the activation of the AKT-CREB axis.

The cells were also treated with the PKA inhibitor H89. As shown in Figure 5C,E,F, H89 markedly suppressed the PAP-1-induced increase in p-Creb1 levels and significantly downregulated the renin levels without altering total Creb1 levels. Immunofluorescence staining confirmed that PAP-1 treatment upregulated the nuclear p-Creb1 signals, which were attenuated upon H89 treatment (Figure 5D,F). These results show that Kv1.3 inhibition in collecting duct-derived cells promotes renin expression through a Ca^2+^-dependent mechanism involving the activation of the AKT and PKA-CREB signaling pathways. Thus, the Ca^2+^-AKT-PKA-CREB signaling cascade acts downstream of Kv1.3.

## 4. Discussion

This study identified a previously unrecognized signaling axis linking Kv1.3 to RAAS activation. Bioinformatic analysis, genetic manipulation, and pharmacological interventions revealed that *Kcna3* deficiency induces hypertension and renal injury by promoting renin expression through a Ca^2+^-dependent AKT-PKA-CREB signaling cascade.

In this study, *Kcna3* expression was not markedly altered under hypertensive conditions. This indicates that *Kcna3* does not contribute to hypertension pathogenesis through transcriptional dysregulation. These findings indicate that Kv1.3 exerts its regulatory effects through the functional modulation of intracellular signaling pathways. This concept highlights an important paradigm in ion channel biology, whereby channel function, rather than expression level, determines physiological outcomes [24,25]. Consistently, the systemic deletion of *Kcna3* resulted in the development of a hypertensive phenotype accompanied by renal structural remodeling and fibrosis. These findings support a critical role for Kv1.3 in maintaining blood pressure homeostasis and renal integrity. Notably, the hypertensive phenotype is unlikely to be secondary to primary cardiac dysfunction, as echocardiographic analysis and circulating cardiac injury markers (CK, CK-MB, LDH, AST) showed no significant differences between WT and *Kcna3*-deficient mice (Supplementary Figure S1). Instead, these data are more consistent with activation of the renal Kv1.3–RAAS axis. Renal injury, which was characterized by glomerular hypertrophy and interstitial fibrosis, is consistent with the chronic activation of the RAAS, a central regulator of blood pressure and kidney function [26,27].

Transcriptomic analysis showed that *Kcna3* deficiency led to significant enrichment of hormone-related pathways, especially those associated with RAAS. renin was the key effector in these pathways. In vivo and in vitro experiments consistently showed that Kv1.3 inhibition or deletion increased renin expression. In addition, circulating angiotensin II was measured in plasma samples collected with EDTA. Although the inclusion of a comprehensive protease inhibitor cocktail would provide more complete protection against ex vivo peptide degradation, the parallel elevations in plasma renin, angiotensin II, and aldosterone, together with the adrenal transcriptomic enrichment of the aldosterone synthesis and secretion pathway, provide internally consistent evidence supporting activation of the systemic RAAS axis. Immunofluorescence analysis indicated that renin upregulation is primarily localized to renal tubular compartments. Additionally, renin expression partially co-localized with Aqp2-positive collecting duct cells, suggesting a potential contribution of the collecting duct to intrarenal RAAS activation [28,29,30]. We acknowledge that plasma Ang II and aldosterone measurements alone are insufficient to fully establish RAAS regulatory mechanisms. However, the convergence of transcriptomic enrichment, hormonal profiling, signaling pathway analysis, and pharmacological validation provides a coherent mechanistic framework. Finally, while our bulk RNA-seq analysis successfully identified the RAAS and steroidogenesis pathways, we acknowledge that this approach lacks single-cell resolution. Future studies employing scRNA-seq combined with ATAC-seq would offer deeper mechanistic insights into the cell-type-specific expression of Kv1.3 and its downstream signaling events. However, given that the primary goal of this discovery study was to establish global pathway-level changes, our bulk RNA-seq data provide a solid foundation and were independently validated by rigorous physiological and pharmacological assays. Therefore, we believe scRNA-seq is a valuable direction for future research.

Importantly, the physiological relevance of CD-associated tubular renin upregulation should be interpreted in the context of systemic versus intrarenal RAAS regulation. To address this, we propose a conceptual framework that, for analytical clarity, distinguishes two functional roles of renin: (1) CD-derived renin acting as an intrarenal amplifier, and (2) JG cell-derived renin driving systemic RAAS activation. However, we acknowledge that these two pools may interact in pathological states. While juxtaglomerular (JG) cells are the primary source of circulating renin, accumulating evidence supports a distinct role for collecting duct (CD)-derived (pro)renin as a local amplifier of the intrarenal RAAS rather than a direct endocrine contributor [31]. CD-secreted (pro)renin can interact with the (pro)renin receptor (PRR) within tubular compartments, promoting localized angiotensin II generation and enhancing sodium and water reabsorption, which are key determinants of long-term blood pressure control [32]. Importantly, CD-derived (pro)renin is generally considered to act locally and does not substantially enter the systemic circulation; rather, the intrarenal Ang II generated via PRR activation promotes sodium and water reabsorption, driving volume expansion and hypertension through a local mechanism [31,33]. This volume-driven pathway provides a functional link between CD renin upregulation and systemic hypertension that is independent of direct renin secretion into the bloodstream. In this framework, the increased CD-associated renin observed in our study is interpreted as an intrarenal mechanism that contributes to hypertension through volume expansion rather than direct entry into the circulation. The persistent elevation of circulating renin and Ang II despite established hypertension requires explanation. Under physiological conditions, elevated blood pressure and increased Ang II suppress JG cell renin release via a negative feedback loop involving intracellular Ca^2+^ oscillations [34]. However, in pathological states such as chronic renal injury and fibrosis, this feedback can be overridden [35]. In our *Kcna3* KO mice, chronic intrarenal RAAS hyperactivation and progressive renal structural remodeling may create local micro-environmental changes—including micro-ischemia and fibrosis—that override classical baroreceptor-mediated suppression of JG cells. Additionally, while our study did not directly measure JG cell calcium dynamics, it is plausible that loss of Kv1.3 may impair the temporal pattern of calcium signaling in JG cells. In JG cells, the inhibitory feedback by Ang II is mediated by intracellular Ca^2+^ oscillations, and disruption of these oscillations, even without changes in average calcium levels, can lead to paradoxical renin release [36]. Given that Kv1.3-mediated K^+^ efflux is critical for maintaining membrane potential and calcium signaling in various cell types, including immune cells and neurons [37], it is conceivable that Kv1.3 deficiency in JG cells could similarly disrupt this regulatory feedback, although this hypothesis requires direct validation in future studies. The concomitant elevation in JG-derived circulating renin levels may thus reflect additional contributions from JG cells, potentially secondary to renal structural remodeling or local microenvironmental changes that override classical baroreceptor-mediated suppression [31]. Taken together, our findings support a model in which (1) CD-derived renin contributes to hypertension through intrarenal volume expansion, and (2) the concomitant rise in circulating renin reflects a distinct JG cell response—potentially driven by local micro-ischemia or direct impairment of calcium-dependent feedback due to Kv1.3 loss—that overrides classical feedback suppression. While our data support a volume-dependent mechanism through CD renin upregulation and PRR-mediated sodium retention, increased intracellular Ca^2+^ in vascular cells may also directly contribute to elevated blood pressure through enhanced vascular tone and peripheral resistance. Thus, the hypertensive phenotype observed in *Kcna3*-deficient mice likely reflects the combined effects of volume-dependent mechanisms (via the RAAS/PRR/ENaC axis) and vascular mechanisms (via increased vascular tone and resistance). Both mechanisms may act together to produce the hypertensive phenotype observed in *Kcna3*-deficient mice. Next, we explored the signaling mechanisms linking Kv1.3 loss to renin upregulation.

Mechanistically, *Kcna3* deficiency disrupts membrane potential and promotes intracellular Ca^2+^ accumulation. This is consistent with previous findings, which demonstrated that Kv1.3 regulates K^+^ efflux and membrane repolarization [22,23,38]. Loss of Kv1.3 activity leads to membrane depolarization, which may promote opening of voltage-gated Ca^2+^ channels. The upregulated intracellular Ca^2+^ functions as a critical second messenger to activate downstream signaling pathways [9,39,40].

This study showed that the upregulated Ca^2+^ activates both PI3K-AKT and cAMP-PKA pathways [41,42], converging on p-Creb1. CREB is a well-established regulator of *REN* transcription. CREB activation provides a mechanistic link between upstream ion channel dysfunction and downstream hormonal regulation [43,44]. Pharmacological inhibition using BAPTA and H89 showed that both Ca^2+^ signaling and PKA activity are required for Creb1 phosphorylation and renin upregulation, establishing a causal signaling axis downstream of Kv1.3. Future studies using chromatin immunoprecipitation (ChIP) assays could further validate the direct binding of CREB to the renin promoter, thereby providing direct evidence for the transcriptional mechanism proposed here. These findings support a model in which Kv1.3 functions as a critical upstream regulator linking membrane excitability to endocrine control of blood pressure. In particular, *Kcna3* deficiency promotes Ca^2+^-dependent activation of AKT and PKA, leading to Creb1 phosphorylation and enhanced *REN* transcription, which results in RAAS activation and hypertension development.

Several limitations of this study should be noted. Although CD-associated renin expression was predominantly upregulated in renal tubular compartments and was partially co-localized with Aqp2-positive regions, the precise cellular source and the relative contribution of different nephron segments were not fully resolved. While intracellular renin expression was consistently increased, renin secretion into the extracellular space or tubular lumen was not directly quantified; therefore, the functional extent of renin release remains to be determined. Direct measurement of renin/prorenin secretion in conditioned media will be an important objective for future functional studies. Future cell-type-specific studies are needed to further define the precise cellular sources of renin and their relative contributions to the hypertensive phenotype [45,46]. We acknowledge that direct measurement of renin activity or concentration was not performed in the current study; future studies incorporating these assays would provide additional functional validation of RAAS activation. We also acknowledge that in vivo RAAS blockade experiments (e.g., with an AT1 receptor blocker or renin inhibitor) were not conducted in the current study; such experiments would provide definitive causal evidence for the role of Ang II in the hypertensive phenotype. Additionally, in vivo pharmacological blockade will be a key priority for our future translational research [47]. Nevertheless, the combined transcriptomic, hormonal, and functional data provide a coherent mechanistic framework that strongly supports a causal role of Ang II in the hypertensive phenotype observed in *Kcna3*-deficient mice. In addition, blood pressure measurements were limited to adult stages (12–14 weeks), and earlier time points were not assessed, preventing precise determination of hypertension onset and progression. Although our 3-week telemetry recordings, including the newly added daytime/nighttime data, demonstrate a stable hypertensive phenotype, longer-term recordings could provide further support for chronic changes; this remains a limitation. In vivo renal functional measurements, such as glomerular filtration rate (GFR) or urinary electrolyte excretion, were not conducted in the current study; these assessments should be addressed in future investigations and could provide additional physiological relevance. Juxtaglomerular (JG) cell-specific investigations were also not performed. It should be noted that our M-1 cell experiments were designed as a proof-of-concept to test the intracellular signaling pathway in a renal epithelial context, rather than to exclude the possibility of similar mechanisms in JG cells. Future studies using JG cell lines, primary JG cell cultures, or JG-cell-specific knockout models are needed to define the relative contributions of these two renin pools. In addition, although the renin antibody used in this study detected a single band (45 kDa), this likely represents total renin-related protein and does not distinguish between prorenin and active renin, which limits interpretation of the functional state of renin. Finally, given the broad expression of Kv1.3, including in immune cells, systemic and immune-mediated effects cannot be fully excluded in the global knockout model [48]. In addition, cardiac immunofluorescence was not performed, although Western blotting confirmed reduced Kv1.3 protein in cardiac tissue. Despite these limitations, the consistency of our findings across in vivo and in vitro models provides strong support for the proposed Kv1.3–Ca^2+^–AKT–PKA–CREB–RAAS signaling axis.

## 5. Conclusions

In summary, this study identifies Kv1.3 as a novel regulator of blood pressure and renal homeostasis. We demonstrate that *Kcna3* deficiency promotes hypertension and renal fibrosis through activation of a Ca^2+^-dependent AKT-PKA-CREB signaling cascade that leads to pathological renin upregulation and is accompanied by systemic RAAS activation. These findings reveal an unrecognized link between membrane excitability and endocrine control of blood pressure. From a translational perspective, targeting Kv1.3 or its downstream signaling components may represent a promising therapeutic strategy for hypertension and associated renal injury. Future studies employing cell type-specific manipulations and in vivo pathway validations are warranted to further refine this mechanism and explore its clinical potential.

## Data Availability

The original contributions presented in this study are included in the article/Supplementary Material. Further inquiries can be directed to the corresponding author.

## Supplementary Materials

The following supporting information can be downloaded at https://www.mdpi.com/article/10.3390/biom16091262/s1, Figure S1: Echocardiographic analysis and circulating cardiac injury biomarkers in WT and *Kcna3* KO mice. Figure S2: Transcriptomic analysis of the renal cortex from *Kcna3* knockout (KO) mice; Figure S3: Transcriptomic analysis of the adrenal gland from *Kcna3* knockout (KO) mice; Figure S4: Daytime and nighttime blood pressure data from telemetry recordings in WT and *Kcna3* KO mice; Figure S5: Immunofluorescence staining of p-CREB and renin expression; Figure S6: Complete image of all original Western blot membranes with molecular weight labels.

## Abbreviations

The following abbreviations are used in this manuscript:

Kv1.3	Potassium voltage-gated channel subfamily A member 3
WKY	Wistar–Kyoto
SHRs	Spontaneously hypertensive rats
BAPTA-AM	1,2-Bis(2-aminophenoxy)ethane-N,N,N′,N′-tetraacetic acid acetoxymethyl ester
H89	N-[2-(p-Bromocinnamylamino)ethyl]-5-isoquinolinesulfonamide
PAP-1	5-(4-Phenoxybutoxy)psoralen
cAMP	Cyclic adenosine monophosphate
GAPDH	Glyceraldehyde-3-phosphate dehydrogenase
KEGG	Kyoto Encyclopedia of Genes and Genomes
H&E	Hematoxylin and eosin
PBS	Phosphate-buffered saline
