# Supplementary material for: Kcna3 Deficiency Promotes Renin-Associated Hypertension Through Ca2+-Dependent AKT–PKA–CREB Signaling
Source: Biomolecules. 2026 Aug 31;16(9):1262. doi: 10.3390/biom16091262 (PMC13604294; doi:10.3390/biom16091262)

Figure 1C

KV1.3-Heart

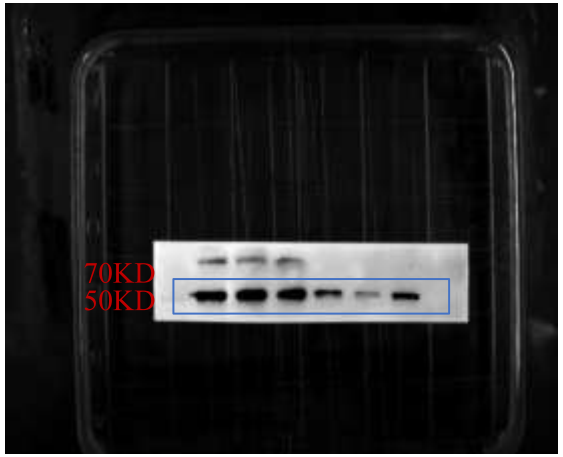

KV1.3-Kidney

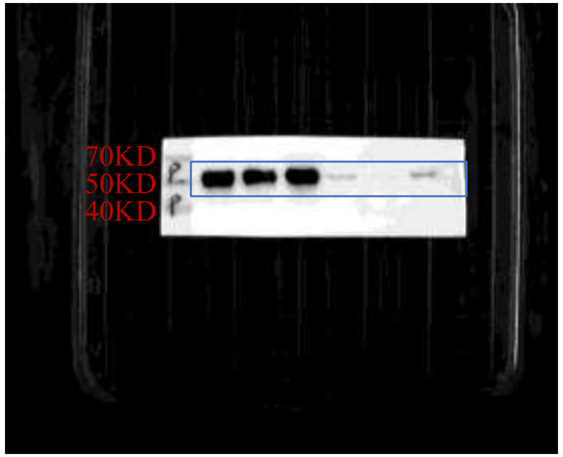

KV1.3-GAPDH

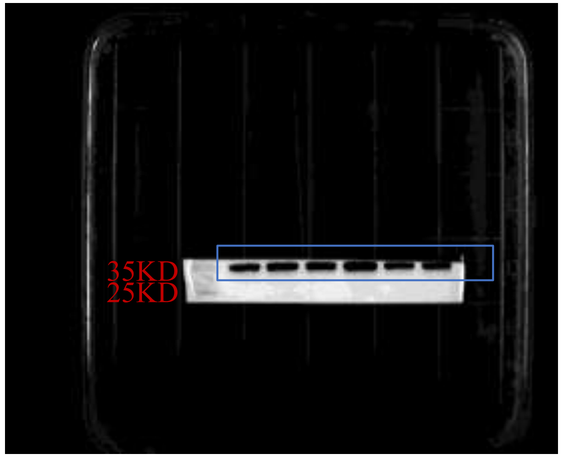

Figure 2G

Mmp2

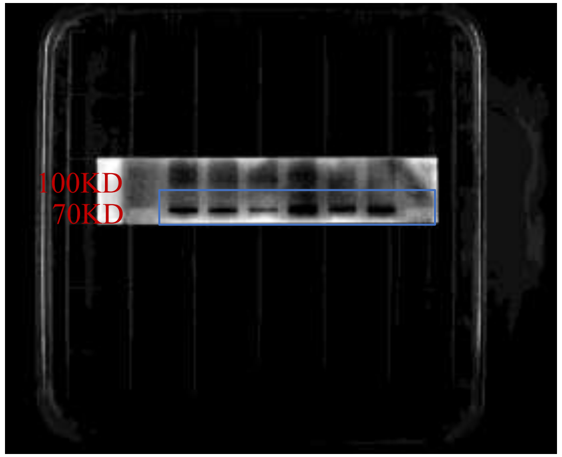

Mmp9

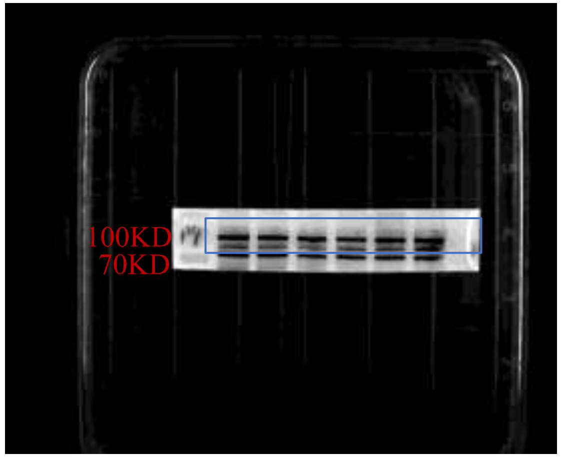

GAPDH

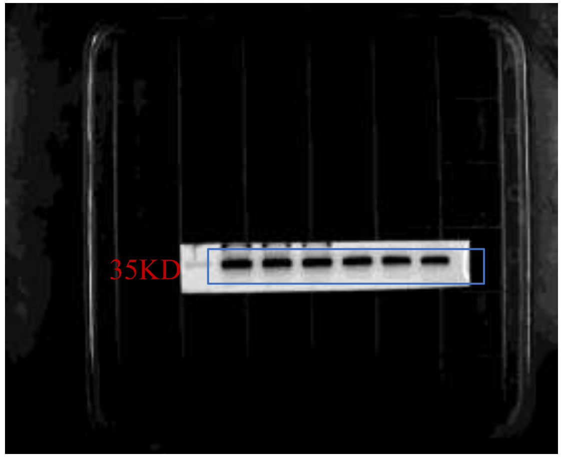

Figure 3G

Renin

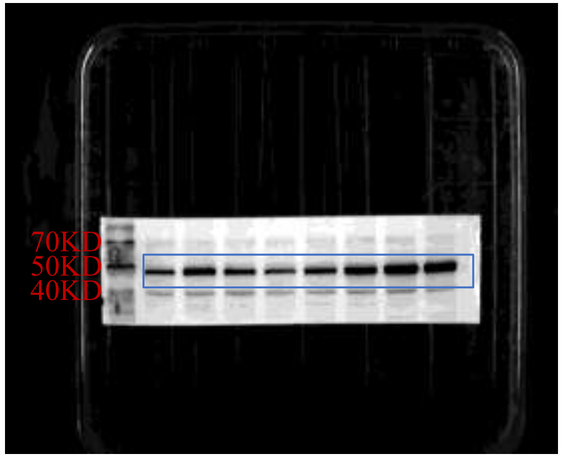

GAPDH

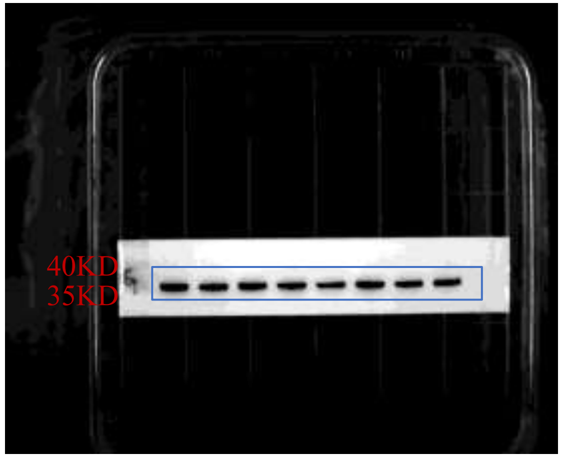

Figure 3H

Renin

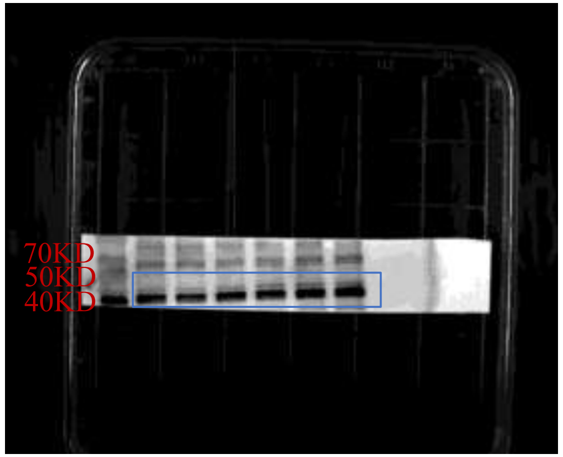

GAPDH

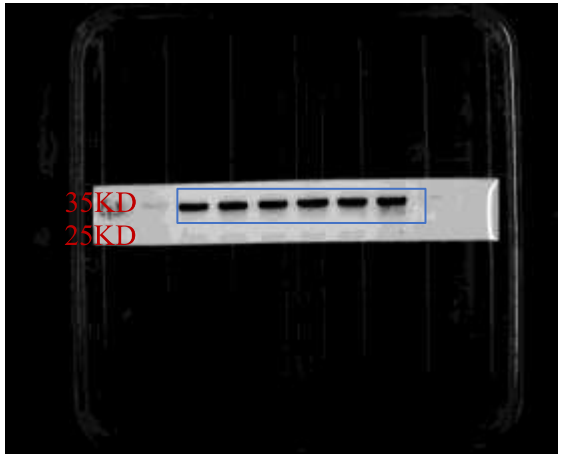

Figure 4D

Pik3ca

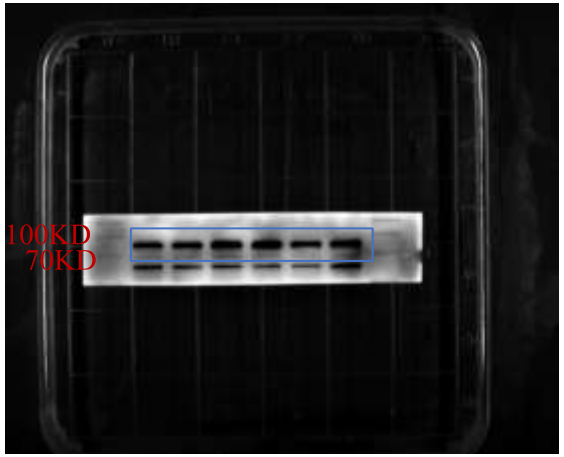

P-Pik3ca

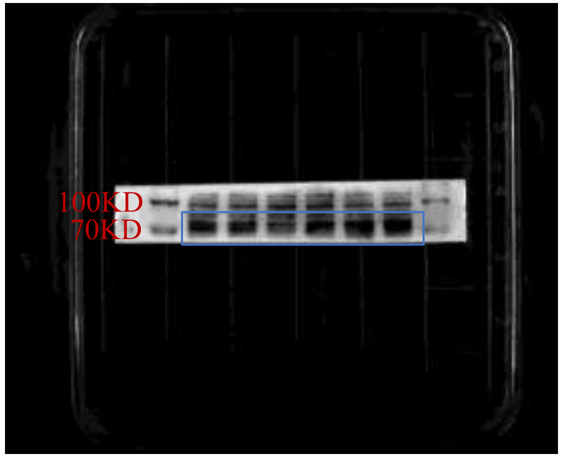

Akt

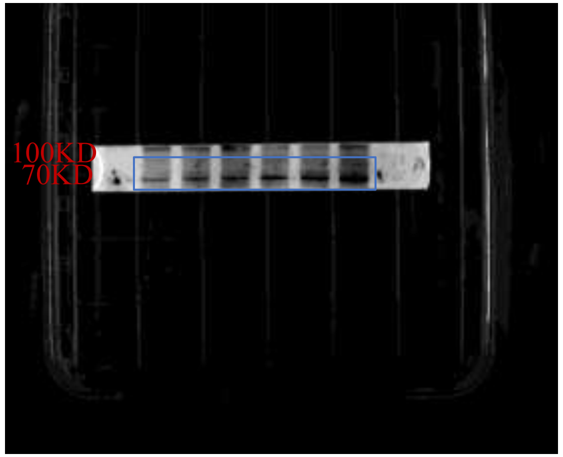

P-Akt

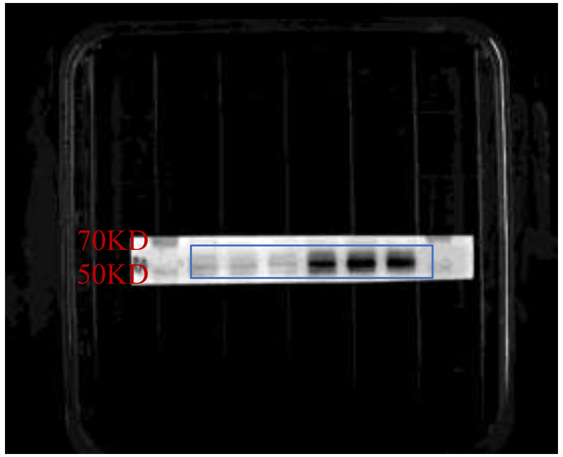

GAPDH

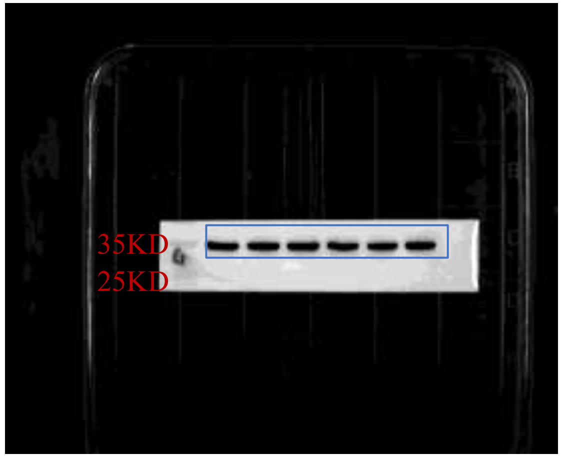

Figure 4F

Creb1

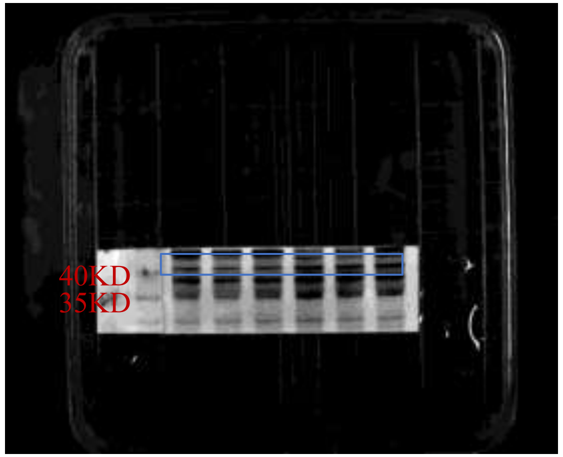

P-Creb1

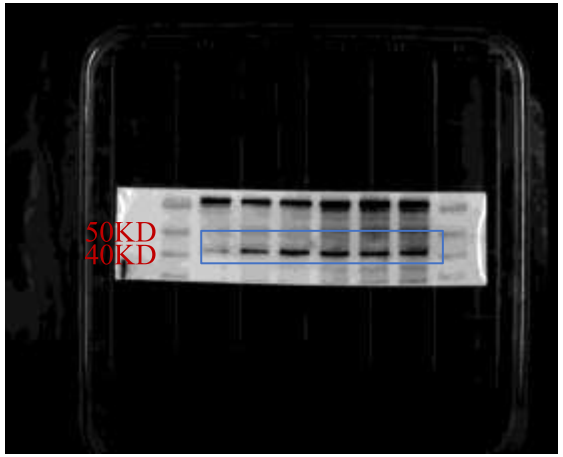

GAPDH

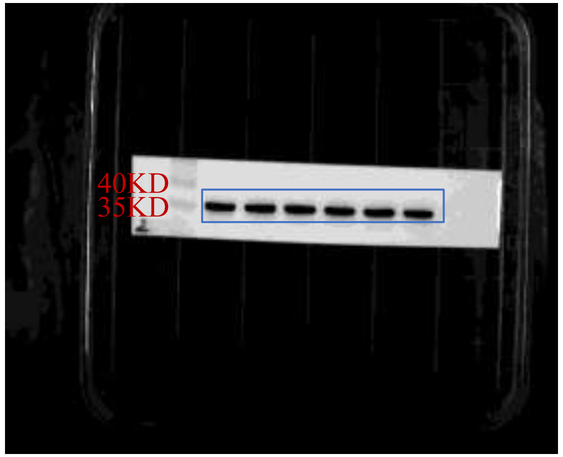

Figure 5A

Akt

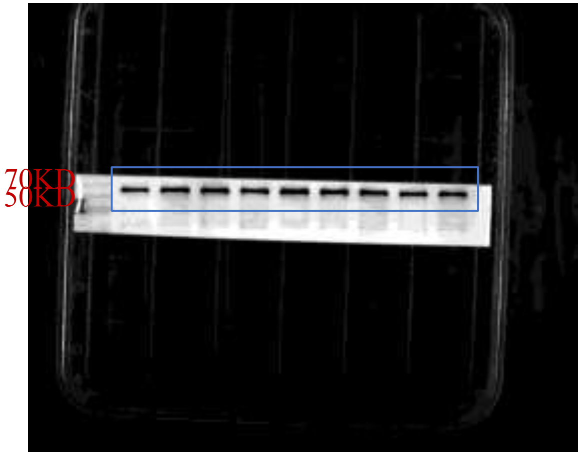

P-Akt

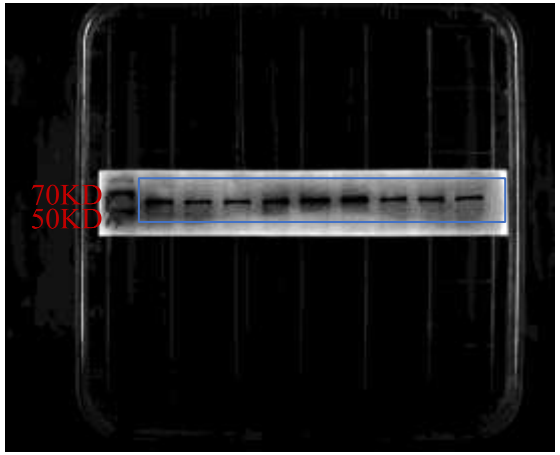

Creb1

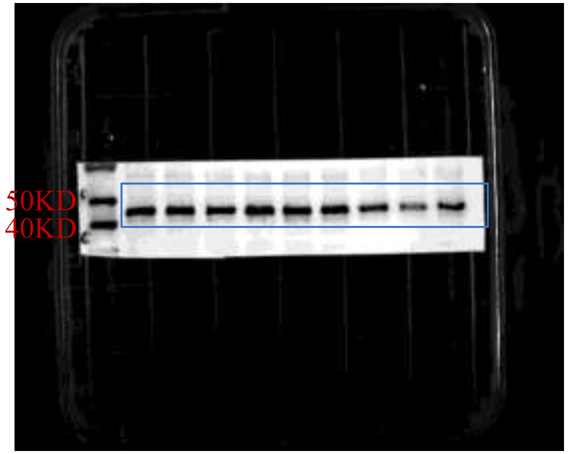

Figure 5A

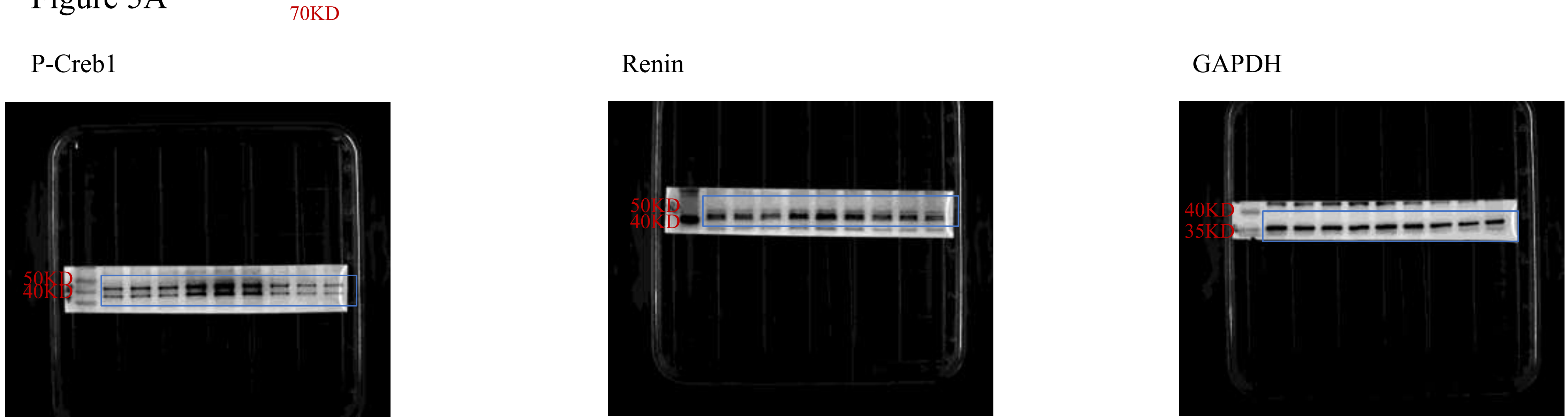

Figure 5C

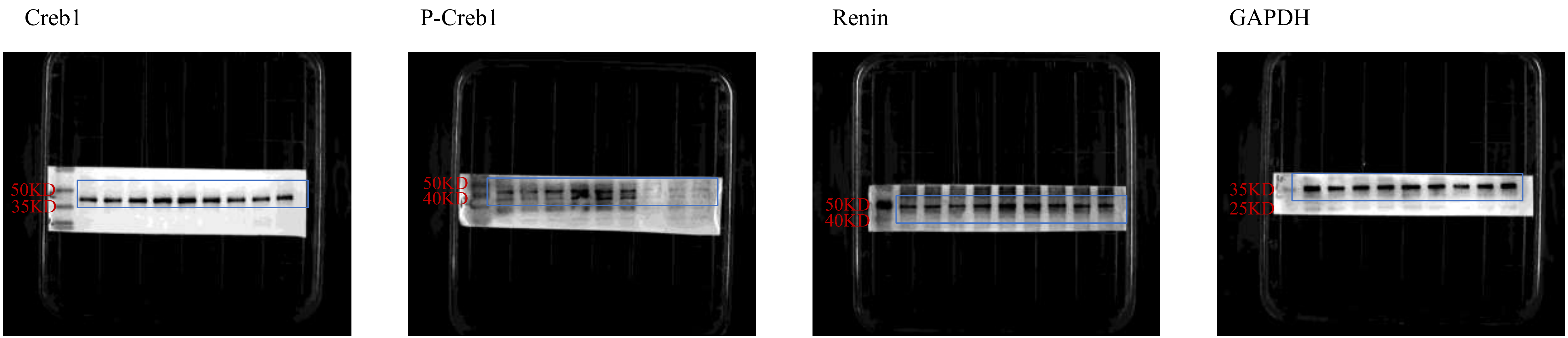

Supplement: Supplementary file 1 [file biomolecules-16-01262-s001.zip › Supplementary Figure S6/Supplementary Figure S6.pdf]
